# “Immunotherapy of cancer: present status and future promise”: Danish Cancer Society Symposium, Copenhagen, Denmark, 23rd–25th September 2013

**DOI:** 10.1007/s00262-014-1585-0

**Published:** 2014-07-24

**Authors:** Marco Donia, Rikke Lyngaa, Sine Reker Hadrup

**Affiliations:** 1 Department of Hematology 65Q9, Center for Cancer Immune Therapy, University Hospital Herlev, Herlev Ringvej 75, 2730 Herlev, Denmark; 2 Department of Oncology, University Hospital Herlev, Herlev, Denmark

**Keywords:** Immune therapy, Cancer therapy, 19th Danish Cancer Society Symposium, T cell therapy, Tumour immunology

## Introduction

The Cancer Society Symposium `Immunotherapy of Cancer—Present Status and Future Promise´ was held at a time when immunotherapy is experiencing a major breakthrough in terms of clinical efficacy and proving its value as an important treatment modality for cancer. A number of highlights from recent findings were presented at the meeting by a selection of international and national speakers. The symposium primarily focused on the following topics: (1) adoptive cell therapy, (2) therapeutic vaccination, (3) cellular regulators in the tumour microenvironment, and (4) targeting immune-regulatory pathways. The Cancer Society Symposium was a small intensive meeting with an educational focus. It hosted 50 young scientists, many of whom were PhD students who presented their own projects as a component of the poster session.

## Adoptive cell therapy

Adoptive cell transfer immunotherapy (ACT) was pioneered by Steven A. Rosenberg and colleagues at the National Institutes of Health, USA, in the early 1990s. When autologous tumour infiltrating lymphocytes (TILs) were infused into patients with metastatic melanoma, this treatment resulted in unprecedented clinical responses. The original protocol, in some cases with minor modifications, was later extended to a handful of other centres, which achieved similar results in small single-institution phase I/II trials. Two of these centres presented their recent findings at this Symposium. **Inge Marie Svane** (Herlev Hospital, Denmark) presented data from an ongoing phase I/II trial where TILs were infused in combination with IL-2 in low (2 MIU/day, sc. for 14 days) or intermediate (“decrescendo-regimen”, 18 MU/m^2^ over 6, 12, 18 and 24 h, followed by 4.5 MU/m^2^/day for 3 days) doses. In the low-dose group of six patients, there was an overall response rate of 33 %, which represented two complete responders. In the intermediate-dose group (15 patients), there was an overall response rate of 53 % (eight patients) with one complete responder. The data presented indicated that reduced doses of IL-2 could be used without diminishing clinical efficacy. Because toxicities related to the administration of high-dose IL-2 have been a major challenge, which previously impaired broad application of these methods, these results can stimulate the initiation of ACT programs at other institutions.

Furthermore, Svane showed that upon recognition of an autologous tumour, IFN-γ/TNF-α-producing T cells were significantly correlated to clinical outcome. Indeed, this finding demonstrated that tumour reactivity is essential and that strategies to increase the fraction of tumour reactive T cells in the final product may have a clinical effect. Consistent with this idea, pre-exposure of autologous tumours to IFN-γ increased overall T cell recognition by TIL products via an up-regulation of HLA molecules. Thus, pre-sensitisation of tumour lesions with interferons could serve as a valuable approach to increase tumour recognition, as presented in a poster session by Marco Donia (Herlev Hospital, Denmark).

Combination therapies may additionally increase the tumour elimination mediated by adoptively transferred TILs, as was further alluded to by **Patrick Hwu** (MD Anderson Cancer Center, Houston, USA). His group is similarly performing adoptive cell therapy with melanoma tumour infiltrating lymphocytes, but they are also very actively investigating combination therapies, including combination with BRAF inhibition, as this targeted therapy has been shown to increase tumour infiltration by T cells. Furthermore, a high-throughput screening platform to identify compounds that most efficiently enhance the ability of tumour-specific T cells to kill autologous tumour and to identify transcripts associated with immune resistance in tumour cells were performed. On the basis of these screens, selected drug candidates could be identified for experimental validation of combinatorial therapy.

**Carl June** (University of Pennsylvania, Philadelphia, PA, USA) showed two different aspects of the potency of adoptively transferred gene-modified T cells. First, he demonstrated the off-target reactivity of a MAGE-A3-directed T cell receptor (TCR) towards the cardiac muscle protein titin, resulting in two cases of fatal cardiac toxicity. This finding clearly showed the overwhelming potency of T-cell-mediated toxicity and highlights the need for efficient strategies to screen for cross-reactivity of TCRs.

The second and more encouraging results regarding gene-modified T cells described the use of chimeric antigen receptors (CARs) directed to CD19 (CAR19). T cells expressing CAR19 have now been successfully transferred to several paediatric and adult patients with acute lymphocytic leukaemia (ALL) and chronic lymphocytic leukaemia (CLL). In ALL, 10 of 12 treated patients achieved a complete response after treatment, with most responses still persisting years after therapy. Thus, the complete response rates were higher in ALL than in CLL, and in a few cases, CLL patients relapsed with a CD19-negative tumour.

Importantly, all patients undergoing CAR19 therapy experienced B cell aplasia and required supportive immunoglobulin treatment. Other significant toxicities included tumour lysis syndrome, cytokine release syndrome, and macrophage activation syndrome. CAR-T cells trafficking to the central nervous system were identified, providing a rationale for the treatment of primary brain tumours with gene-engineered T cells.

Thus, these results illustrated that specific T cells under suitable circumstances are very efficient in killing target-expressing cells. The selection of targets and the specificity of the CAR or TCR in use are essential to avoid serious adverse events, and for more heterogeneous tumours, simultaneous targeting of several molecules is needed to avoid target-negative escape mutants.

## Therapeutic vaccination

Therapeutic vaccination in cancer has long struggled to induce clinical efficacy and to correlate the induced immune reactivity against vaccine antigens to clinical efficacy. A recent study from Immatics Biotechnologies GmbH, Tübingen, Germany, presented by **Harpreet Singh**, elegantly showed that both of these challenges could be overcome. He presented data from a phase I study and a subsequent phase II study with renal cell carcinoma patients receiving a therapeutic vaccination against multiple tumour-associated peptides. These peptides were identified based on the MHC-associated presentation on renal cell carcinoma tissues when compared to healthy tissue. Interestingly, the data suggested an important role for preconditioning with cyclophosphamide (300 mg/m^2^, single infusion) to induce clinically relevant immune responses because the preconditioned treatment group with one or more immune responses showed better survival than those that were not preconditioned; however, they harboured the same number of immune responses. The number of regulatory T cells was decreased following cyclophosphamide treatment. Furthermore, overall survival appeared to be associated with a low number of circulating myeloid-derived suppressor cells (MDSCs) and high serum concentrations of CCL17 and APOA1, in which the latter of the two was particularly evident in combination with cyclophosphamide. Immatics is currently working towards the generation of personalised vaccines, with a selection from “off-the-shelf” peptides based on the immunogenicity and tumour expression determined on a patient-specific basis.

Several other examples showed that peptide vaccination might also be effective in a therapeutic setting. Most evidently, this was demonstrated in high-grade human papillomavirus (HPV)-positive vulvar intraepithelial neoplasia with a clinical outcome associated with lesion size at entry, and the induction of a strong vaccine prompted HPV-specific immune response and the absence of a vaccine-induced regulatory response. At the symposium, **Sjoerd van der Burg** (Leiden University Medical Centre, The Netherlands) presented data from a recent phase II clinical trial involving 20 patients with HPV16-positive recurrent cervical cancer. The vaccine, which consisted of long overlapping peptides covering the entire sequence of E6 and E7 onco-proteins, administered in Montanide ISA-51, failed to induce a clinical response with an effect on overall survival. However, among the vaccinated patients, the long-term survivors (patients surviving longer than the median overall survival rate of 12.6 months) stimulated a better immune response to vaccination than the short-term survivors (patients surviving less than 12.6 months), when measured based on HPV16-specific proliferation or IFN-γ ELISPOT. These HPV-targeting vaccines also served to pioneer long-peptide vaccinations, a strategy that was also applied by **Gustav Gaudernack** (Oslo University Hospital, Norway), as a means to stimulate immune responses against hTERT. They observed that longer survival correlated with epitope spreading, including more than two peptides from hTERT.

**Graham Pawelec** (University of Tübingen, Germany) suggested that the mechanism behind epitope spreading could be related to the in vitro functionality of antigen-specific T cells, which correlated with long-term survival of late-stage melanoma patients. Thus, the presence of circulating NY-ESO-1- or Melan-A-responsive T cells was correlated with prolonged survival, but this was counteracted by a high level of circulating MDSCs. Furthermore, for Melan-A but not NY-ESO-1 responses, a negative effect of CD4 T cell reactivity was observed when these cells produced either IL-4 or IL-17, which potentially represent a non-classical form of regulatory T cell.

Another major component in therapeutic vaccination consists of dendritic cell-based vaccinations. Dendritic cells (DCs), which are major players in the initiation of an immune response, have been extensively explored as vaccine candidates. Their source of origin and in vitro maturation methods used have resulted in a large variety of DCs being used in clinical trials, but there is still no consensus as to what is the best DC type for vaccination purposes. **Kris Thielemans** (Free University of Brussels, Belgium) has pioneered the use of RNA-electroporated DCs for vaccine purposes. Immature monocyte-derived DCs were electroporated with mRNA encoding CD40L, CD70, and caTLR4 (TriMix mRNA). In a clinical trial with 35 advanced melanoma patients, the DCs were also electroporated with gp100, tyrosinase, MAGE-C2, or MAGE-A3 mRNA encoded as a fusion with HLA class II targeting signal of DC-LAMP (TriMixDC-MEL). This DC-based vaccine was administered either intradermally (ID) or combined ID with intravenous (IV) administration. These results indicated that combined IV/ID administration increased clinical efficacy, with two partial responses (PR) and two complete responses (CR) observed out of 15 patients in this cohort. The group further tested the use of TriMixDC-MEL in combination with ipilimumab treatment. Here, five CR and five PR were observed among 35 evaluable patients, demonstrating that such combinations may increase the clinical responses observed with either therapy alone.

**Glenn Dranoff** (Dana Farber Cancer Institute, Boston, USA) elegantly showed how a proinflammatory microenvironment might drive lung cancer pathogenesis. Subsequently, he showed his recent results on the use of autologous melanoma cells engineered to secrete GM-CSF as the main vaccine component, administered with or without anti-CTLA4 antibodies. He observed that the ratio of CD8 T cells to FoxP3-positive regulatory T cell correlated to tumour regression, which suggested that this balance might be further skewed to increase a clinical response. In-depth analyses of patients with clinical benefits from this therapy and evaluation in experimental mouse models have led to a target discovery program, identifying factors associated with immune-mediated tumour destruction. In addition, he showed how T cell-targeted immunotherapy can efficiently trigger a potent humoral response that modulates the dynamics of the tumour microenvironment.

## Cellular regulators in the tumour microenvironment

Infiltration of T cells in human tumours may have great prognostic value, as demonstrated most significantly in colorectal cancer via the mapping of immune involvement, as pioneered by **Jérôme Galon** (INSERM, Paris, France). In a large interdisciplinary effort, he described the effect of various immune cell infiltrates, numbers, location, and interaction in correlation with clinical outcome. Galon and colleagues analysed the immune landscape of colorectal cancer in correlation with progression-free survival. They determined cell types associated with good clinical outcome (e.g. CD3, CD20, CD8, CD45RO) when present either at the invasive margin or at the centre of the tumour. Other cell types have a negative effect on survival, e.g. IL-17-producing cells, and, interestingly, this is primarily evident when they are located at the invasive margin. Furthermore, the group reported the interplay between all of the investigated cell types and specifically emphasised the role of the B cell component and its correlation with high expression of CXCL13 (B cell chemoattractant), CD20, and IL-21 with a favourable clinical outcome.

A simplified description of the immune infiltrate, the immunoscore, has been shown to outrank the TNM classification in terms of prognostic value. The prognostic value of the immunoscore is currently being evaluated at several independent centres.

One mechanism by which tumours modify immune infiltration and immune function is via altered myelopoiesis, which causes an accumulation of immune-suppressive cells. Factors regulating this accumulation have been studied by **Vincenzo Bronte** (University of Verona, Italy) and colleagues. Bronte presented data at the symposium demonstrating that down-regulation of microRNA miR-142-3p promoted the differentiation of bone marrow cells into M2-type macrophages. Furthermore, miR-142-3p affects IL6-mediated promotion of macrophage generation. Identification and targeting of such mechanisms responsible for the induction of the immune-suppressive tumour microenvironment may improve the efficacy of cancer immune therapy.

In addition, microRNAs of the miR200 family are also involved in regulating cancer-associated fibroblasts (CAFs) and macrophages of the M2 phenotype, as presented by **Pedro Barcellos-de-Souza** (University of Florence, Italy). CAFs and M2 macrophages have been demonstrated to exhibit strong immune-suppressive capabilities, which play a crucial role in tumour progression.

In general, microRNAs appear to play an important role in the immunosuppressive microenvironment, and in the immunosuppressive phenotype of many cancer cells. Cancer cells often escape immune attack via a down-regulation of HLA or changes in the antigen-processing machinery. These defects may be driven either by structural alterations or by deregulation. **Barbara Seliger** (University of Halle, Germany) described how these processes are similarly regulated by microRNA. Thus, identification of regulatory miRs may provide both prognostic and therapeutic value.

Another subgroup of immune-suppressive cells, MDSCs, has been shown to display a strong inhibition of T cell-mediated antitumour activities via the production of nitric oxide and arginase. As shown by **Viktor Umansky** (DKFZ, Heidelberg, Germany), this cell type may be targeted through ultralow (non-cytotoxic) doses of chemotherapy, a strategy that can easily be implemented in combination with various immune therapeutic treatments.

One mechanism by which MDSC-like cells are induced in the tumour microenvironment was presented by **Rolf Kiessling** (Karolinska Institute, Stockholm, Sweden). He showed that melanoma cells may instruct CD14-positive monocytes to become MDSC-like cells with potent T cell inhibition properties. This conversion was dependent on cyclooxygenase-2 (COX-2) production and activation of the STAT-3 signalling pathway. This mechanism provides targets for alleviating the T cell suppression in the tumour microenvironment.

## Targeting immune-regulatory pathways

Mechanisms of tumour-induced immune suppression/ignorance may be divided into two categories, as shown by analyses of melanoma metastases presented by **Thomas F. Gajewski** (University of Chicago, USA). Some patients/lesions demonstrate an inflamed phenotype characterised by T cell infiltration that is functionally impaired through PD-L1, indoleamine-pyrrole 2,3-dioxygenase (IDO) and Treg interactions and associated with T cell anergy. Other patients/lesions display a non-inflamed phenotype, which is characterised by a lack of T cell infiltration. The mechanism underlying the non-inflamed phenotype is still incompletely understood, but it is correlated with the lack of innate immune signalling, the absence of a type I IFN signature, and low chemokine production. In addition, tumour-derived vascular endothelial cells are most likely distinct, as evidenced by the differential expression of the endothelin B receptor. As many non-inflamed lesions showed high arginase expression, MDSCs might be a component associated with T cell exclusion. These differential mechanisms are clinically important. In patients with T cell infiltration, PD-L1, IDO, and Tregs are largely seen as counteractive mechanisms for antitumour immune responses, and these patients are more likely to respond to CTLA-4, PD1/PD-L1 or IDO blockade therapy. Other strategies should be explored in patients with a non-inflamed phenotype to initiate innate immune activation and to promote endogenous T cell priming and recruitment into the tumour microenvironment.

Therapeutic targeting of immune-regulatory mechanisms has already initiated a new era for immune therapy for cancer. Evidently, patients can be cured by halting their intrinsic immune inhibitory pathways. Thus, spontaneous antitumour immune responses have the ability to eradicate a growing tumour when the tumour-induced inhibitory mechanisms are abolished. This has clearly been demonstrated through the clinical implementation of anti-CTLA4 and anti-PD1 antibodies. Recent data from a clinical evaluation of these compounds were presented by **Antoni Ribas** (UCLA, Los Angeles, USA). Impressive clinical responses were observed with PD1 and CTLA-4 blockade, which presented objective response rates of 38 and 11 %, respectively. The combination of these two agents displayed an objective response rate of 53 % in a phase I trial, including 53 patients who received concurrent therapy with nivolumab and ipilimumab and 33 patients who received sequenced treatment, which enhanced enthusiasm in the field to investigate combinatorial therapy.

A potential source for combinatorial therapy is BRAF-targeted therapy. Inhibition of mutated BRAF via vemurafenib results in significant clinical responses; however, the duration is usually limited. Molecular characterisation of the drug-related effects on signalling pathways has revealed a paradoxical effect in BRAF wild-type cells, resulting in the activation of the MAPK signalling pathway. This activation results in increased in vivo cytotoxic activity and intratumoural cytokine secretion by adoptively transferred T cells when combined with BRAF inhibition. This further resulted in superior antitumour reactivity when combinatorial therapy was investigated in immunocompetent mice. As more targeted therapies are emerging, the field for combining targeted therapies with immunotherapies will likely rapidly expand. These combinations may enhance immunotherapy without the potential risk of T cell killing, which forms an intrinsic threat for combinations with chemotherapy.

An alternative strategy for targeting inhibitory pathways is the capacity of the immune system to strike back on most regulatory circuits. It has been described and was presented by **Mads Hald Andersen** (Herlev University Hospital, Denmark) that cytotoxic T cells against immune inhibitory molecules, such as IDO, PD-L1 and FoxP3, exist and may be enhanced in combination with other tumour-associated antigens to increase tumour cell killing. This strategy is ideal for combinations with therapeutic vaccination, and it was recently demonstrated in a small phase I study with IDO peptide vaccination of 15 HLA-A2-positive stage II–IV non-small cell lung cancer (NSCLC) patients that vaccination against IDO may support long-lasting disease stabilisation.

## Final remarks

On the basis of the data presented during this meeting, the future for cancer immunotherapy looks very promising—numerous new strategies and combination therapies are emerging, and the number of clinical trials within this field is rapidly evolving. Through in-depth molecular characterisation of these clinical initiatives, we will likely gain knowledge of the molecular mechanisms and signatures that are predictive for treatment outcome and provide a basis for rational design and patient selection to increase response rates.

This meeting provided an exceptional opportunity for young scientists approaching the field of cancer immunotherapy to interact with established leaders who presented their most recent works in an informal and stimulating environment. Compelling Q&A sessions extended discussions on recent results, initiated enthusiastic debates between speakers and students regarding the future of immunotherapy and generated novel ideas, which anticipate the next steps forward towards a cure for patients affected by metastatic malignancies.

## Abbreviations

ACT: Adoptive cell transfer immunotherapy
ALL: Acute lymphocytic leukaemia
CAF: Cancer-associated fibroblast
CAR: Chimeric antigen receptor
CAR19: Chimeric antigen receptors directed to CD19
CLL: Chronic lymphocytic leukaemia
COX-2: Cyclooxygenase-2
CR: Complete response
DC: Dendritic cell
HPV: Human papillomavirus
ID: Intradermally
IDO: Indoleamine-pyrrole 2,3-dioxygenase
IV: Intravenous
MDSC: Myeloid-derived suppressor cell
NSCLC: Non-small cell lung cancer
PR: Partial responses
TCR: T cell receptor
TIL: Tumour infiltrating lymphocyte

